# Refractive shift of silicone oil tamponade in pseudophakic eye

**DOI:** 10.1186/s12886-016-0243-z

**Published:** 2016-08-16

**Authors:** Wei Fang, Jiuke Li, Xiaohong Jin, Jing Zhai, Yuanmin Dai, Yumin Li

**Affiliations:** Ophthalmology Department of SIR RUN RUN SHAW hospital, SIR RUN RUN SHAW Institute of Clinical Medicine of Zhejiang University, #3 Qingchun East Road, Hangzhou, Zhejiang 310016 People’s Republic of China

**Keywords:** Refractive shift, Silicone oil tamponade, Theoretical formula, Artificial lens

## Abstract

**Background:**

Refraction change of silicone oil (SO) tamponade in phakic and aphakic eye has been studied thoroughly; however, it is rarely studied in pseudophakic eye. In this paper we aimed to deduce a theoretical formula predicting refractive shift of silicone oil tamponade in pseudophakic eye and compared it with clinical findings.

**Method:**

A theoretical formula was deduced through strict geometric optical principles under the Helmholtz Schematic eye model. Pre/postoperative refractive status of patients who previously underwent phacoemulsification, intraocular lens (IOL) implant, vitrectomy, SO tamponade and required SO extraction was studied.

**Results:**

Twenty-six patients (27 eyes, 13 males and 13 females) were studied. Refractive error of SO-off was -1.88 ± 2.73D, and of SO-in was 2.02 ± 3.90. Refractive shift of SO tamponade was -3.90 ± 1.74D. Refractive shift was significantly associated with refractive power of IOL (*r* = -0.7903, *p* < 0.0001, Pearson correlation test) and anterior chamber distance (ACD, *r* = 0.3840, *p* = 0.0480, Pearson correlation test). Theoretical refractive shift was -4.10 ± 1.51D, and there was no significant difference between the theoretical and the clinical refractive shift (*p* = 0.3329, Paired T test).

**Conclusions:**

Refractive shift of SO tamponade in pseudophakic eye correlates with refractive power of implanted IOL and ACD, and strong correlation between theoretical formula and clinical findings was detected.

**Electronic supplementary material:**

The online version of this article (doi:10.1186/s12886-016-0243-z) contains supplementary material, which is available to authorized users.

## Background

Refraction change of silicone oil (SO) tamponade in phakic and aphakic eye has been studied thoroughly; however, it is rarely studied in pseudophakic eye. Phacoemulsification, artificial lens implantation combined with vitrectomy and silicone oil (SO) tamponade has been widely applied in rhegmatogenous and or tractional retinal detachment [1, 2]. In some cases SO may not be extracted for a long time or even for ever because of high risk of retinal detachment, such as acute retinal necrosis syndrome [3–6]. So predicting degree of refractive shift of SO tamponade in these pseudophakic eyes is useful. Though posterior surface radius of intraocular lens (IOL) has proved to be significantly associated with this refraction change, practically it is not convenient to acquire this parameter from manufactures and calculate the refractive shift for many physicians [7, 8]. So here we chose other easier obtained parameters to deduce a theoretical formula predicting the refraction change following strict geometric optical principles, and compared it with clinical findings.

## Methods

### Theoretical formula deduction

Helmholtz Schematic eye is used to deduce the theoretical formula. In this model the eye’s refractive system is considered a compound system consisting of two refractive systems: cornea (a single surface) and IOL (a thin lens with two surfaces). Refractive index (RI) of aqueous humor and vitreous fluid is 1.336. The intraocular lens is presumed to be a biconvex lens with equal curvature on both sides. The theoretical total eye refractive power is derived from the single surface power formula and compound lens calculation formula under the condition of SO tamponade or not.

Single surface power formula:

$$ \mathrm{D}\kern0.5em =\kern0.5em \frac{{\mathrm{n}}_1-{\mathrm{n}}_2}{\mathrm{r}} $$

D: refractive power of a single surface n_1_/n_2_: RI of substance at two sides of the single surface r: radius of refractive surface

Total refractive power of a thin lens:

$$ \mathrm{D}\kern0.5em =\kern0.5em {\mathrm{D}}_1\kern0.5em +\kern0.5em {\mathrm{D}}_2 $$

D: total refractive power D_1_/D_2_: refractive power of the lens’s two surfaces

Refractive power derivation of a compound or thick lens:

$$ \mathrm{D}\kern0.5em =\kern0.5em {\mathrm{D}}_1+{\mathrm{D}}_2-\frac{\mathrm{d}}{\mathrm{n}}{\mathrm{D}}_1{\mathrm{D}}_2 $$

D: total refractive power of a compound system D_1_/D_2_: refractive power of two single systems d: distance between two systems n: refractive index of substance between two systems

From the three formulas above, a theoretical formula of the total pseudophackic refractive power can be derived:

$$ {\mathrm{D}}_0\kern0.5em =\kern0.5em {\mathrm{D}}_{\mathrm{c}}+{\mathrm{D}}_{\mathrm{i}}\kern0.5em -\kern0.5em \frac{\mathrm{d}}{{\mathrm{n}}_{\mathrm{h}}}\ {\mathrm{D}}_{\mathrm{c}}{\mathrm{D}}_{\mathrm{i}}\kern0.5em =\kern0.5em {\mathrm{D}}_{\mathrm{c}}+\left(\frac{{\mathrm{n}}_{\mathrm{i}}-{\mathrm{n}}_{\mathrm{h}}}{{\mathrm{r}}_{\mathrm{i}\mathrm{a}}}+\frac{{\mathrm{n}}_{\mathrm{i}}-{\mathrm{n}}_{\mathrm{v}}}{{\mathrm{r}}_{\mathrm{i}\mathrm{p}}}\right)\kern0.5em -\kern0.5em \frac{\mathrm{d}}{{\mathrm{n}}_{\mathrm{h}}}\kern0.5em {\mathrm{D}}_{\mathrm{c}}\left(\frac{{\mathrm{n}}_{\mathrm{i}}-{\mathrm{n}}_{\mathrm{h}}}{{\mathrm{r}}_{\mathrm{i}\mathrm{a}}}+\frac{{\mathrm{n}}_{\mathrm{i}}-{\mathrm{n}}_{\mathrm{v}}}{{\mathrm{r}}_{\mathrm{i}\mathrm{p}}}\right) $$

D_0_: total refractive power of a pseudophakic eye D_c_/D_1_: refractive power of cornea and IOL n_v_/n_i_/n_h_: RI of vitreous fluid, artificial lens and aqueous humor r_ia_/r_ip_: anterior and posterior radius of IOL d: distance from cornea to anterior surface of IOL (ACD)

When presumed unchanged corneal curvature and ACD after SO injection, a formula of theoretical refractive power with SO completely filled can be as follows:

$$ {\mathrm{D}}_1\kern0.5em =\kern0.5em {\mathrm{D}}_{\mathrm{c}}+\left(\frac{{\mathrm{n}}_{\mathrm{i}}-{\mathrm{n}}_{\mathrm{h}}}{{\mathrm{r}}_{\mathrm{i}\mathrm{a}}}+\frac{{\mathrm{n}}_{\mathrm{i}}-{\mathrm{n}}_{\mathrm{so}}}{{\mathrm{r}}_{\mathrm{i}\mathrm{p}}}\right)\kern0.5em -\kern0.5em \frac{\mathrm{d}}{{\mathrm{n}}_{\mathrm{h}}}\ {\mathrm{D}}_{\mathrm{c}}\left(\frac{{\mathrm{n}}_{\mathrm{i}}-{\mathrm{n}}_{\mathrm{h}}}{{\mathrm{r}}_{\mathrm{i}\mathrm{a}}}+\frac{{\mathrm{n}}_{\mathrm{i}}-{\mathrm{n}}_{\mathrm{so}}}{{\mathrm{r}}_{\mathrm{i}\mathrm{p}}}\right) $$

D1: total refractive power of a pseudophakic eye with SO completely filled n_so_: RI of silicone oil

From these two formulas we can derive the refractive shift formula from SO unfilled to completely filled:1

If the IOL is presumed to be a biconvex lens with equal curvature on both sides, then a function of refractive power and RI of IOL can substitute for r_ip_ (from the single surface power formula):

$$ {\mathrm{r}}_{\mathrm{i}\mathrm{p}}\kern0.5em =\kern0.5em \frac{2\left({\mathrm{n}}_{\mathrm{i}}-{\mathrm{n}}_{\mathrm{h}}\right)}{{\mathrm{D}}_{\mathrm{i}}} $$

So the theoretical formula can be derived as:

When we substitute constants for some variables (n_v_ = 1.336, n_so_ = 1.403, n_h_ = 1.336), we obtain:2

In this formula we can infer that refraction change of SO tamponade in pseudophakic eyes theoretically depends on IOL power, RI, ACD and corneal refractive power.

### Clinical observations

Patients who underwent uneventful phacoemulsification, IOL implant, vitrectomy, SO tamponade and required SO extraction by one experienced surgeon were enrolled in this study, including highly myopic eyes. Exclusion criteria were unstable fundus (such as macular edema, macular pucker, retinal hemorrhage, or macular hole occurred after SO extraction), obviously SO emulsification obstructing optometry examinations, vitreous cavity incompletely filled, posterior capsule rupture, corneal opacity and incomplete clinical data. Silicone oil extraction was performed under inferior-temporal 23G pars plana incision and superior 20G incision. Both incisions were sutured after surgery. Refractive errors before and after SO extraction were confirmed by retinoscopy under dilated pupil and best spectacles correction. Corneal refractive power (D_c_) was measured by corneal topography (ATLAS 9000,Carl Zeiss, Germany) before and after operation (postoperative D_c_ was used in theoretical calculation). Anterior chamber depth (ACD) was measured through ultrasonic A scan (E-Z Scan AB5500+, Sonomed, USA) after SO extraction, while preoperative ACD was not collected because of acoustic interference of some centrally accumulated emulsified silicone particles during supine examination. Axis length was detected pre/postoperatively by IOL master (Carl Zeiss, Germany). Six types of IOL were implanted in this patients group (Akreos adapt, Baush & Lomb, USA, RI 1.458; Akreos AO, Baush & Lomb, USA, RI 1.458; Softec HD, Lenstec, USA, RI 1.458; Tecnis ZCB00, AMO, USA, RI 1.470; Tecnis ZA9003, AMO, USA, RI 1.470; AR40e, AMO, USA, RI 1.470) and all patients were injected with 5000CS SO (RT SIL-OIL 5000, Carl Zeiss, Germany, RI 1.403). All the postoperative data were collected on the second day after surgery. Pearson linear correlation analysis was applied in correlation of refractive shift and selected variables. Paired T test was used to compare theoretical refractive shift with clinical result. All significant levels were designated as 0.05. All of those analyses were performed by an independent analyzer with software SAS 9.2. We declared that this study is in accordance with the declaration of Helsinki, approved by SIR RUN RUN SHAW hospital ethic committee and all patients have signed an informed consent, receiving no stipend.

## Results

**Fig. 1:**
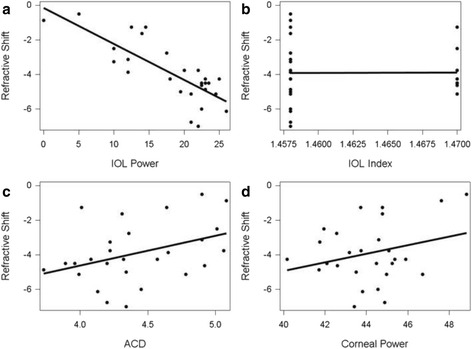
Correlation of refractive shift of SO tamponade with selected variables (Pearson correlation test). **a** Refractive shift vs. IOL power(*r* = -0.7903, *p* < 0.0001). **b** Refractive shift vs. IOL index (refractive index 1.458 versus 1.470, *r* = 0.0058, *p* = 0.9773). **c** Refractive shift vs. ACD (anterior chamber distance, *r* = 0.3840, *p* = 0.0480). **d** Refractive shift vs. corneal power(*r* = 0.2696, *p* = 0.1738)

**Fig. 2:**
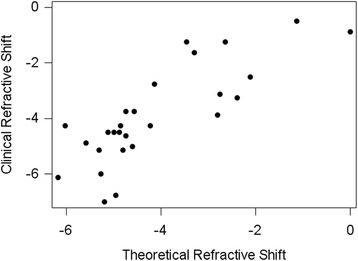
Comparison of refractive shift with theoretical one. No significant difference between theoretical and clinical refractive shift was observed. (*p* = 0.3329, Paired T test)

There were 26 patients (27 eyes) from Dec. 2009 to Sep. 2014 at Ophthalmology Department of Sir Run Run Shaw Hospital enrolled in this study, 13 males and 13 females. Refractive power of IOL implanted ranged from 0 Diopters (D) to 26D. ACD after SO extracted was 4.42 ± 0.39 mm (M ± SD). Corneal power was 43.96 ± 1.99D before and 44.15 ± 1.90D after SO extracted respectively (*p* = 0.0137, Paired T test). Corneal power increment was 0.20 ± 0.38D. Pre/postoperative axis length was 25.84 ± 3.28 mm, 25.85 ± 3.27 mm, respectively, without significant change (*p* = 0.9762, Paired T test). Refractive error of SO-off was -1.88 ± 2.73D, and SO-in was 2.02 ± 3.90. Refractive shift of SO tamponade was -3.90 ± 1.74D, which was significantly associated with refractive power of IOL (*r* = -0.7903, *p* < 0.0001, Pearson correlation test) and ACD (*r* = 0.3840, *p* = 0.0480, Pearson correlation test) (Fig. 1). Theoretical refractive shift was -4.10 ± 1.51D, according to the theoretical formula above. There was no significant difference between the theoretical and the clinical refractive shift (*p* = 0.3329, Paired T test, Fig. 2).

## Discussions

In deduction of this theoretical formula, we presumed all the other variables selected (like D_c_, ACD) were unchanged before and after SO extraction (except substitution of SO for vitreous fluid), but practically the corneal power increased slightly after operation [9–11]. In this study this increment was about 0.20D, namely 0.45 % changes on the cornea. Many authors also reported corneal curvature slightly increased after three port par plana vitreoretinal surgery, especially when the scleral incisions were sutured [10, 11]. However this increment did not have obvious affect on the total refractive power of the compound refractive system and the refractive shift because of the negligible changes on the corneal power. In this study we chose the postoperative D_c_ to calculate the theoretical refractive shift according to the theoretical formula.

Çalik B et al reported ACD did not obviously change in a week after SO tamponade and pars plana vitrectomy, but it did increase between one week and one month [12]. We could not acquire the exact preoperative ACD parameter because of the acoustic interference of some SO particles in anterior chamber during supine ultrasonic A scan in this study. So here we calculate the refractive shift of SO tamponade early after SO extraction to maximally reduce errors from ACD changes.

IOL power depends on RI of materials and curvature of both surfaces. For clinical calculative convenience, we presume that the IOL in this study is a biconvex lens with equal radius on both sides. But actually, one IOL in the six kinds implanted has an asymmetrical design (Akreos AO, anterior surface slight steeper than posterior one). So it should be taken into account when use this formula clinically.

Song WK et al. revealed that the refraction changes of SO tamponade in pseudophakic eye significantly correlated with posterior radius and RI of artificial lens [7]. We also found this correlation in the theoretical formula ①, however it is not convenient to get this parameter practically, so we surrogate this variable with much easier obtained one, namely IOL refractive power. Meanwhile, not exactly corresponding to the theoretical formula, we did not find significant correlation of refractive shift with IOL RI and corneal curvature during clinical observation, maybe due to the relatively small patient group in this study, which should be studied further.

As mentioned above, our main limitation in this study is the sample size, though a strong correlation is found between theoretical and clinical data. We recommend further studies with larger sample size to obtain more precious formulas.

## Conclusions

Refractive shift of SO tamponade in pseudophakic eye correlates with refractive power of implanted IOL and ACD, and strong correlation between theoretical formula and clinical findings was detected. Future correction formula based on IOL power and ACD can be simple and predictable method through further studies with larger sample size.

## Abbreviations

ACD, anterior chamber distance; CI, confidence interval; D_0_/D_1_/D_c_/ D_i_, refractive power of pseudophakic eye without and with SO filled, cornea and intraocular lens; IOL, intraocular lens; M, mean; RI, refractive index; r_ia_ /r_ip_, anterior and posterior radius of intraocular lens; SD, standard deviation; SO, silicone oil.

## Additional files

Additional file 1: Original data of this study. (XLS 74 kb) Additional file 2: Certification of medical ethnic committee. (JPG 72 kb)
