# Supplementary figures and images for: Refractive shift of silicone oil tamponade in pseudophakic eye
Source: BMC Ophthalmol. 2016 Aug 16;16:144. doi: 10.1186/s12886-016-0243-z (PMC4986366; doi:10.1186/s12886-016-0243-z)

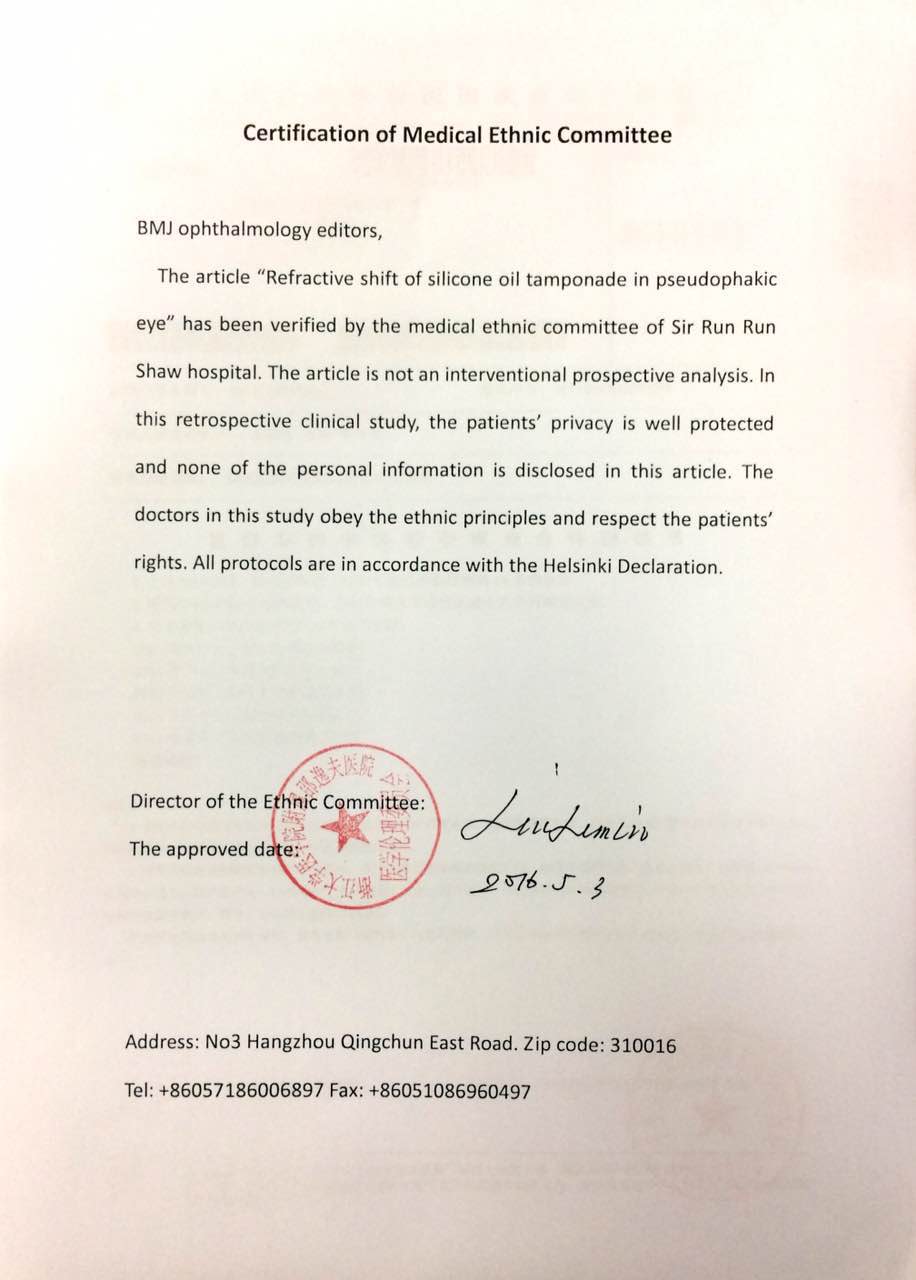

Supplement: Additional file 2: — Certification of medical ethnic committee. (JPG 72 kb) [file 12886_2016_243_MOESM2_ESM.jpg]
